# Advancing gender equity in gynecology and obstetrics: perspectives from the German workforce

**DOI:** 10.1007/s00404-025-08167-4

**Published:** 2025-08-27

**Authors:** Jasmin Neuhold, Frauke von Versen-Höynck

**Affiliations:** 1 Department of Obstetrics and Gynecology, Hannover Medical School, Carl-Neuberg-Strasse 1, 30625 Hannover, Germany; 2 Gynecology Research Unit, Hannover Medical School, Carl-Neuberg-Strasse 1, 30625 Hannover, Germany

**Keywords:** Gender equity, Leadership disparities, Diversity, Academic medicine, Germany

## Abstract

**Purpose:**

Despite the high proportion of women in medical education and clinical training, female physicians remain underrepresented in leadership and academic positions in Gynecology and Obstetrics in Germany. This study investigates structural and cultural factors contributing to this disparity and highlights trends in workforce composition and recognition in the field.

**Methods:**

We conducted a descriptive, cross-sectional analysis using publicly available data from the German Federal Statistical Office and the German Medical Association (2004–2024), as well as database from the German Society for Gynecology and Obstetrics (DGGG).

**Results:**

In 2023, women comprised 73% of gynecologists in Germany. Female representation declined with advancing career stage. In 2024, 82% of resident physicians at German university hospitals were women, while they held 26% of department head positions in Gynecology and Obstetrics. Female membership in the DGGG reached 75%, with 40% on the executive board. In 2022, 65% of major awards went to men, while women received most poster and lecture prizes (61%) at the biannual conference.

**Conclusions:**

While gender diversity in the clinical workforce has improved, systemic inequities persist in academic and leadership domains. Addressing these requires structural reforms, increased transparency in recruitment and promotion processes, and targeted programs supporting women’s professional advancement. Enhanced visibility, mentorship, and inclusive institutional policies are essential to ensure gender-equitable development in the specialty.

## What does this study adds to the clinical work

**Table:** 

This study highlights the persistent underrepresentation of women in leadership and academic positions in Gynecology and Obstetrics despite their majority in the workforce. Addressing structural barriers and promoting targeted career support are crucial to fully harness the potential of female physicians in the field.	

## Introduction

In recent years, awareness of the importance of gender equity has significantly increased, particularly in professional and academic fields. It is well established that balanced gender representation enhances workplaces productivity [1]. In science, diverse research teams tend to achieve better outcomes, producing more inclusive studies that address gender-specific needs—a critical factor in overcoming the historical disadvantages faced by women in health care [2].

Medicine reflects similar trends. For over 2 decades, more than half of the medical students in Germany have been women, and the proportion of female physicians has been steadily increased [3, 4]. Evidence suggests that female physicians achieve better patient outcomes compared to their male counterparts [5].

Despite this progress, women face challenges in advancing their academic careers. Although they often obtain doctorates early, their publication rates remain lower [6]. The underrepresentation of women in leadership positions persists due to structural, institutional, and cultural barriers which continue to hinder women´s progress in medicine and science [7]. Recent studies have highlighted the persistent barriers to career advancement for women in gynecological societies, despite an increase in female representation in the workforce [8, 9].

This discrepancy underscores the significant role of professional societies and policymakers in fostering gender equity. Notably, the German Society for Gynecology and Obstetrics (Deutsche Gesellschaft für Gynäkologie und Geburtshilfe e.V., DGGG) has committed to promoting career development and improving the work–life balance for its members [10].

Achieving true gender equity in Gynecology and Obstetrics—a field predominantly comprised of women—requires a data-driven dialog about current conditions and the effectiveness of implemented measures.

This work aims to provide a comprehensive overview of the status of gender equity in this field in Germany. By examining progress and challenges, we seek to lay the groundwork for new initiatives and strategies to support the next generation of female physicians.

## Methods

### Data collection

The empirical foundation for this study is primarily based on data of the German Federal Statistical Office (Bundesamt für Statistik), the German Medical Association (Bundesärztekammer), and the DGGG [4, 11].

Various anonymous data from the Federal Statistical Office on medical students in Germany, habilitations in Gynecology and Obstetrics, and working time models in Gynecology and Obstetrics in German hospitals were sorted and evaluated by gender. Data from the German Medical Association were also analyzed within the relevant categories, such as age structure, board certification, inpatient care, and out-patient care, regarding their development over the past decade. The DGGG administrative office provided data about their members and conferences.

Furthermore, the websites of 36 Departments of Gynecology and Obstetrics of German university hospitals and 683 general hospitals were used to research the number of female and male physicians. Specifically, at university hospitals, we discriminated between the different career levels. It is important to note that not all websites contained all the requested data. At the time of retrieval, four university hospital websites and nine general hospital websites did not contain the complete information. In particular certain career levels were absent.

### Definitions

Career levels at university hospitals were defined as residents, board-certified physicians, junior attending physicians, attending physicians, senior attending physicians, vice heads of departments, and heads of departments.

The DGGG (German Society for Gynecology and Obstetrics) is the national professional society representing physicians in the field. The DGGG statutes stipulate that the executive board must be elected every two years by the general meeting. The executive board is the entity that fulfills the role of management and consists of the president, vice president, second vice president (former president), secretary, and treasurer. The DGGG extended board supplements the executive board and includes ten elected representatives from various working and interest groups.

### Outcomes

The primary outcome was the gender distribution across different career levels in Gynecology and Obstetrics in Germany, including board certification, hospital leadership positions, membership and leadership in the DGGG, and participation in conferences and awards. Secondary outcomes included trends over time in these categories.

### Study methodology

This is a descriptive study using publicly available and anonymized data. We present descriptive data organized by gender and visualized through graphs created using Microsoft 365 Excel and Microsoft 365 PowerPoint.

### Statistical analyses

Results are reported as total numbers or percentages, as appropriate.

### Ethical approval

Ethical Committee approval was not required, as no human or animal subjects were involved and data used for analysis were anonymized.

## Results

To examine gender equity in Gynecology and Obstetrics in Germany, a series of comparisons were conducted within the framework of bibliometric analyses. First, the proportion of female and male physicians in the field was compared, followed by a comparison within the professional society for Gynecology and Obstetrics.

### Workforce representation

**Fig. 1:**
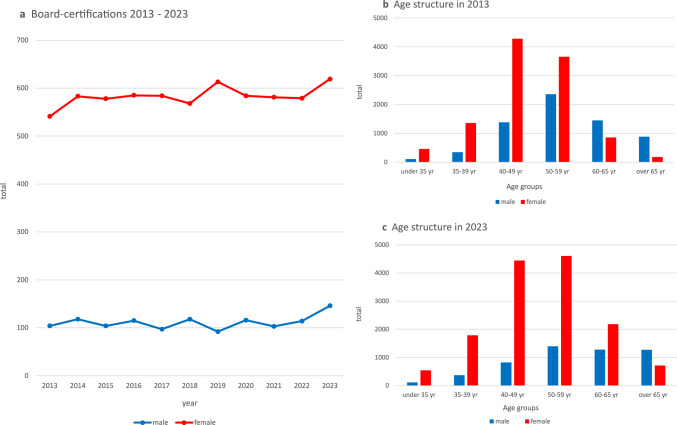
Gender distribution among annual board certifications in Gynecology and Obstetrics in Germany (2013–2023) (**a**), age structure of board-certified German gynecologists and obstetricians by gender in 2013 (**b**), and in 2023 (**c**)

In 2023, the representation of women in the field of Gynecology and Obstetrics reached a new high of 73%. The proportion of women earning board certifications has remained stable at around 80% for the past 15 years (Fig. 1a) [4].

In 2013, women were overrepresented in all age groups under 60 and underrepresented in those over 60 (Fig. 1b). By 2023, there was an increase in female representation in all age groups, reaching 82% in physicians under 35 years and 63% in those aged 60–65, while the over-65 group was dominated by men (83%) (Fig. 1c).

**Fig. 2:**
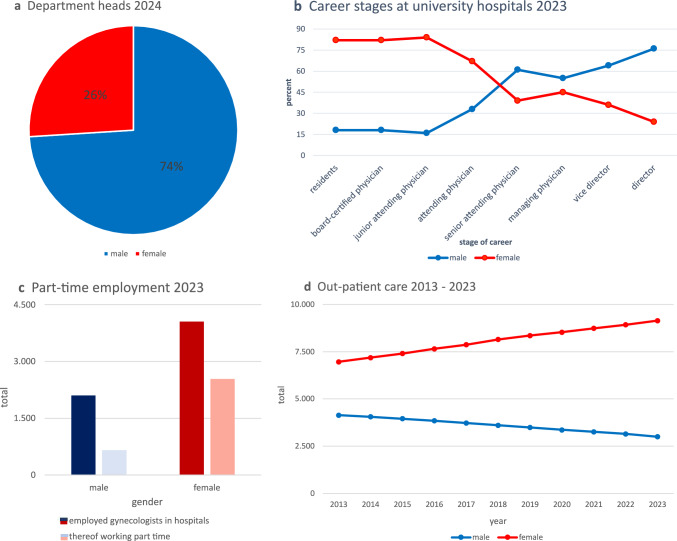
Gender distribution in clinical care in Gynecology and Obstetrics. Department head positions in German 683 general hospitals in 2024 (**a**), female representation at German university hospitals (**b**), part-time employment in general hospitals in 2023 (**c**), and gender distribution in out-patient care from 2013–2023 (**d**)

In 2024, women held 26% of head positions in the respective departments of German hospitals (*n* = 683) (Fig. 2a**)**. At German university hospitals (*n* = 36), the proportion of female physicians was high among residents (82%), board-certified physicians (82%), and junior attending physicians (84%) but declined at more advanced career stages—attending physicians (67%), senior attending physicians (39%), and vice heads of departments (36%)—reaching its lowest among heads of departments (24%) (Fig. 2b).

In 2023, a total of 3195 (52%) physicians working in Gynecology and Obstetrics at German hospitals were employed part-time, with women accounting for 80% of them. Notably, 62% of female gynecologists worked part-time, compared to only 31% of their male counterparts [23] (Fig. 2c). In out-patient care, the proportion of female gynecologists rose significantly from 6,095 (57%) in 2009 to 9,144 (75%) in 2023 (Fig. 2d).

### Representation in the national society

**Fig. 3:**
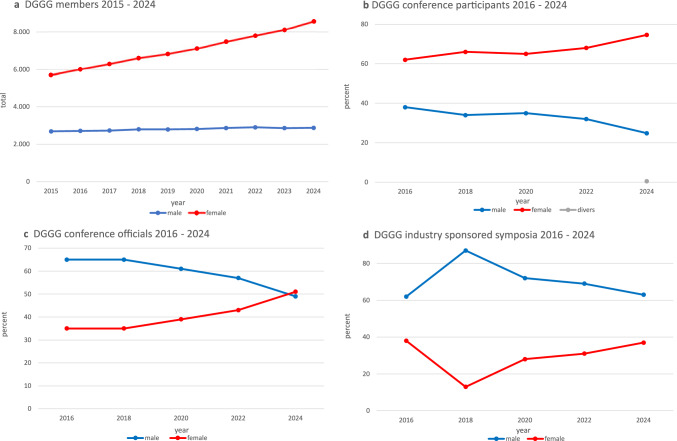
Gender distribution among DGGG members (2015–2024) (**a**), DGGG conference participants 2016–2024 (**b**), conference officials 2016–2024 (**c**), and DGGG conference speakers at industry-sponsored symposia 2016–2024 (**d**)

Over the past two decades, total memberships of the DGGG have more than doubled increasing by 271% from 4,211 in 2004 to 11,438 in 2024. Female membership rose significantly from 1731 (41%) to 8554 (75%), while male membership remained relatively stable, rising from 2,480 in 2004 to 2878 in 2024 (Fig. 3a). According to the administrative office of the DGGG in February 2025, women represented the highest proportion of members in early and mid-career stages, such as residents (90%), board-certified physicians (88%), and senior attending physicians (74%), but were less represented in leadership roles: department heads in general hospitals (27.5%) and department directors of university hospitals (22%).

Under the statutes adopted in September 2015, the DGGG board has been elected five times [12]. In two terms, the first president was female, and in three terms, male. The executive board has consistently included three men (60%) and two women (40%). The highest proportion of women in the extended board was 50% (2018–2020; 2022–2024), in the other terms women accounted for 40% (Table 1).

**Tab 1:** DGGG president, executive board members, and extended board members by gender since 2016

Periods	President		Executive board		Extended board	
			(including the President)			
	Male	Female	Male	Female	Male	Female
2016–2018	0	1	3	2	6	4
2018–2020	1	0	3	2	5	5
2020–2022	1	0	3	2	6	4
2022–2024	0	1	3	2	5	5
2024–2026	1	0	3	2	6	4

Every two years, the DGGG organizes the largest German-speaking congress for Gynecology and Obstetrics. The proportion of female participants rose from 62% in 2016 to 75% in 2024 (Fig. 3b). The share of women in key roles (session chairs, moderators, speakers, and course leaders) increased from 35% in 2016 to 51% in 2024 (Fig. 3c). However, the representation of women in industry-sponsored symposia was consistently lower with the highest proportion of women recorded at 38% (2018) (Fig. 3d) [13–17].

**Fig. 4:**
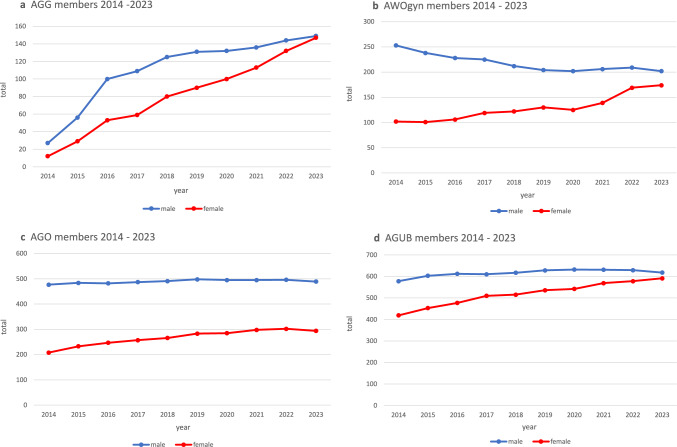
Gender distribution among AGG members (**a**), AWOgyn members (**b**), AGO members (**c**), and AGUB members (**d**) between 2014–2023

The DGGG features a variety of working groups that focus on diverse specialty areas within Gynecology and Obstetrics. The Obstetrics and Prenatal Medicine Working Group (AGG- Arbeitsgemeinschaft für Geburtshilfe und Pränatalmedizin in der DGGG e.V.), established in 2014, initially comprised 27 men (69%) and 12 women (31%). By 2023, the proportion of women had increased to 50% (Fig. 4a). In Aesthetic, Plastic, and Reconstructive Surgical Procedures in Gynecology Working Group (AWOgyn—AG für ästhetische, plastische und wiederherstellende Operationsverfahren in der Gynäkologie e.V.), the percentage of women has also risen significantly over the past decade, from 29% in 2014 to 46% in 2023 (Fig. 4b). The Gynecological Oncology Working Group (AGO—Arbeitsgemeinschaft Gynäkologische Onkologie e.V.) has seen the lowest female representation rising from 30% (2014) to 38% (2023) (Fig. 4c). In the Urogynecology and Pelvic Floor Plastic Reconstruction Working Group (AGUB—Arbeitsgemeinschaft für Urogynäkologie und plastische Beckenbodenrekonstruktion e.V.), the gender distribution has nearly equalized by 2023, with men at 51% and women at 49% (Fig. 4d).

### Representation in research and science

**Fig. 5:**
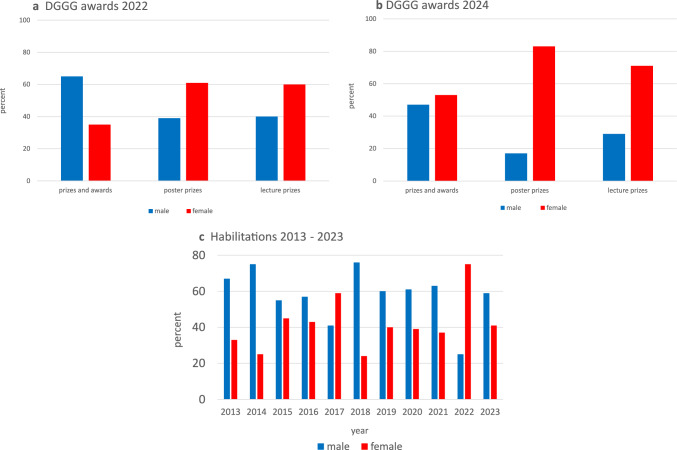
Gender distribution among awards and prizes at the DGGG congress in 2022 (**a**), and in 2024 (**b**), and habilitation trends by gender in Gynecology and Obstetrics 2013–2023 (**c**)

At DGGG congresses, various awards are presented to recognize outstanding contributions to the field. In 2022, female physicians received 35% of the major awards, 61% of the poster prizes, and 60% of the lecture prizes (Fig. 5a). In 2024, the trend continued with women winning 53% of the major awards, 83% of the poster prizes, and 71% of the lecture prizes (Fig. 5b).

An analysis of habilitations over the past two decades revealed that men have consistently outnumbered women in obtaining this qualification except in 2022 (Fig. 5c) [18].

## Discussion

This study analyzes gender equity in Gynecology and Obstetrics in Germany, showing that despite women’s majority in training and early career stages, they remain underrepresented in senior leadership and academia.

Data from the German Federal Statistical Office and the German Medical Association reveal a notable increase in the proportion of female physicians in Gynecology and Obstetrics, with women accounting for 73% of gynecologists in 2023, surpassing the national average of 50% across all specialties [3, 5]. Female board certifications have remained stable at around 80% for the past 15 years [4].

However, while female representation is high at entry and mid-career levels, the situation at senior career stages presents a more complex picture. In 2024, women held 26% of head of department positions in general hospitals and 24% in university hospitals. This decline can be partially attributed to the high proportion of female gynecologists working part-time—80% of part-time physicians in the field are women, compared to only 31% of male gynecologists working part-time in hospitals. Although part-time status alone cannot fully explain the underrepresentation, it does limit career progression opportunities, particularly in management positions, where full-time availability is often prioritized [11].

At this point, it is important to note that while women hold 50% of full-time positions, studies indicate that they are less likely to be offered management roles, especially after becoming mothers or taking on family responsibilities. This is influenced by both male bias and the “queen bee” effect, where women may be less likely to promote other women [19, 20].

The DGGG has witnessed a remarkable surge in female membership, increasing from 41% in 2004 to 75% in 2024. While this reflects the growing presence of women in the field, their representation in DGGG working groups remains capped at 50%.

Despite statutory requirements, women remain underrepresented on the Executive Board (40%) and reached 50% on the Extended Board in only two terms, this level remains insufficient for adequate representation. It should be noted that the representatives of the working groups are selected by the groups themselves through an internal application and election process. The underrepresentation of women may, in part, be attributed to a lower number of female applicants. Structural barriers and self-selection continue to limit advancement into decision-making roles.

The representation of women in research and scientific achievement within Gynecology and Obstetrics is another area of concern. While the increasing proportion of women at lower career levels suggests a thriving community of female researchers, their recognition through awards remains limited. Industry-sponsored symposia have consistently favored male speakers, while women more often receive poster prizes. The phenomenon gender award gap is documented in the literature [21, 22]. The DGGG has found that women are less likely to apply for advertised awards and studies proved that they more often decline speaking invitations or give shorter presentations [23, 24]. This phenomenon is further compounded by the dearth of female role models in the scientific community.

Habilitation data show progress: in 2022, 75% of habilitations were awarded to women, yet over the past two decades, men have achieved this qualification at higher rates [18]. While women publish fewer articles than men at lower career stages, output increases substantially at the clinic director level [6]. Women often achieve academic titles earlier, yet still face challenges in obtaining the highest qualifications [6, 18].

The pursuit of gender equity and the establishment of equitable career opportunities have become critical considerations across various specialized medical disciplines. Other specialties in Germany, such as Cardiology and Plastic Surgery, show even lower female leadership rates [25, 26]. Conversely, the field of Gynecology and Obstetrics exhibits a comparatively higher proportion of women in managerial roles.

Despite the encouraging trends in female participation in Gynecology and Obstetrics, significant challenges remain, particularly regarding leadership and scientific recognition. The DGGG has implemented several initiatives to address these barriers, such as the “Junior-meets-Senior-Lunch” program, which facilitates networking between junior and senior physicians. However, these efforts have not yet sufficed to eliminate the gender disparities that continue to exist in management and research roles.

To address these inequalities sustainably, it is essential to consider the specific needs of women in the workplace. A recent study highlights that the availability of adequate, flexible childcare significantly influences an individual’s choice of employer, regardless of gender. Additionally, the study found that over 70% of women reject long-term employment opportunities in hospitals due to the conflicts between family and career obligations [27].

Job-sharing models, though rare in medicine, have been implemented successfully and may help balance clinical duties and leadership roles [28, 29]. A reevaluation of career progression and leadership roles is also necessary, particularly in academic medicine, where structural and systemic changes are crucial to ensure that women thrive in both clinical and academic leadership.

A key strength of this study lies in its comprehensive approach, combining current data from national sources with targeted literature to provide a multi-level analysis of gender equity in Gynecology and Obstetrics in Germany. However, the analysis is limited by the availability and granularity of publicly accessible data. As the study focuses on a single country and specialty, findings may not be fully generalizable to other contexts or disciplines.

## Conclusion

Despite rising female representation, leadership gaps persist. Addressing them requires systemic changes, flexible structures, mentorship, and unbiased hiring to unlock women’s potential.

## Data Availability

Research data are available from the corresponding author upon reasonable request.
